# The Antioxidant and Anti-Fatigue Effects of Rare Ginsenosides and γ-Aminobutyric Acid in Fermented Ginseng and Germinated Brown Rice Puree

**DOI:** 10.3390/ijms251910359

**Published:** 2024-09-26

**Authors:** Shiwen Feng, Tao Li, Xinrui Wei, Yifei Zheng, Yumeng Zhang, Gao Li, Yuqing Zhao

**Affiliations:** 1 Key Laboratory of Natural Medicines of the Changbai Mountain, Ministry of Education, College of Pharmacy, Yanbian University, Yanji 133002, China; 15104023121@163.com (S.F.); 2022001111@ybu.edu.cn (T.L.); zhengyifei9912@163.com (Y.Z.); 2 College of Functional Food and Wine, Shenyang Pharmaceutical University, Shenyang 110016, China; 18803329203@163.com (X.W.); zym122908@126.com (Y.Z.)

**Keywords:** ginseng, germinated brown rice, fermented puree, rare ginsenoside, antioxidant, anti-fatigue

## Abstract

γ-aminobutyric acid (GABA) and rare ginsenosides are good antioxidant and anti-fatigue active components that can be enriched via probiotic fermentation. In this study, ginseng and germinated brown rice were used as raw materials to produce six fermented purees using fermentation and non-fermentation technology. We tested the chemical composition of the purees and found that the content of GABA and rare ginsenoside (Rh_4_, Rg_3_, and CK) in the puree made of ginseng and germinated brown rice (FGB) increased significantly after fermentation. The antioxidant activity of the six purees was determined using cell-free experiments, and it was found that FGB had better ferric-ion-reducing antioxidant power (FRAP) and 1,1-diphenyl-2-picryl-hydrazyl (DPPH) free radical scavenging rates, exhibiting better antioxidant effects. We then evaluated the antioxidant effect of FGB in HepG_2_ cells induced by H_2_O_2_ and found that FGB can reduce the generation of reactive oxygen species (ROS) in HepG_2_ cells and increase the membrane potential level, thereby improving oxidative damage in these cells. In vivo experiments also showed that FGB has good antioxidant and anti-fatigue activities, which can prolong the exhaustive swimming time of mice and reduce the accumulation of metabolites, and is accompanied by a corresponding increase in liver glycogen and muscle glycogen levels as well as superoxide dismutase and lactate dehydrogenase activities. Finally, we believe that the substances with good antioxidant and anti-fatigue activity found in FGB are derived from co-fermented enriched GABA and rare ginsenosides.

## 1. Introduction

The fast pace of modern life means that people are often in a state of fatigue [1,2]. Fatigue can lead to memory loss, low work efficiency, drowsiness, and lack of energy, and can even cause certain diseases [3,4,5]. The health and safety risks associated with fatigue have received widespread attention [6]. There are many reasons for the onset of fatigue, including the consumption of energy-poor sources and the overproduction and accumulation of metabolic products [7]; immune system disorders and the excessive production of reactive oxygen species (ROS) [8]; and the imbalance of blood oxygen concentrations, in which the glycogen homeostasis of muscle and liver is difficult to maintain [9]. Relief from fatigue has become an important research topic in various countries. The current methods of relieving fatigue include sleep supplements, drugs, and nutritional supplements [10], with the latter becoming one of the main options for alleviating physical fatigue due to increasing health problems.

Whole grains are gaining popularity due to their high nutritional value and bioactive compounds involved in protection against chronic diseases and symptoms such as fatigue [11]. Brown rice (BR) contains germ and bran, which are richer than white rice and contain phytochemicals and nutrients important to health. The use of germination treatment can improve the digestibility and absorption of BR, which can effectively improve its edible and nutritional value [12]. Germinated brown rice (GBR) puree contains a large number of active ingredients, such as γ-aminobutyric acid (GABA), which has a good effect on improving physical fatigue caused by exercise and has attracted considerable attention because of its biological activity [13]. In recent years, the development of GBR functional drinks has flourished, and various GBR drinks with different production processes have appeared on the market [14]. It is worth mentioning that GBR functional drinks, as an emerging healthy puree, are expected to become a significant area of research for the development of anti-fatigue nutritional supplements.

Scientific compatibility is one of the most important technologies for the development of nutritional supplements. As a traditional Chinese medicine, ginseng has been used throughout Asia for many years [15]. The health benefits of ginseng, which are supported by scientific research, include replenishing vital energy, maintaining heart rate, alleviating deficiencies, nourishing the spleen, lungs, and blood, calming nerves, and improving intelligence. It can also stimulate immune function [16] and relieve pain and anxiety [17]. Based on the history and safety of ginseng consumption, it has been listed as a new food resource for use in health food processing. Ginseng contains ginsenoside, polysaccharides, and volatile oils, of which ginsenosides are the main active substances with confirmed efficacy in treating fatigue caused by various factors. The ginsenoside Rb_1_ can reduce the expression of inflammatory factors and affect the activity of indoleamine 2,3-dioxygenase (IDO), and has a good effect on postoperative and exercise fatigue [18]. Rg_1_ can also increase liver and muscle glycogen levels and significantly prolong the exhaustive swimming time of mice [19]. After hydrolysis, ginsenosides can be converted into rare ginsenosides, such as Rg_3_, Rh_4_, and CK [20]. In recent years, there have been many studies on the use of rare ginsenosides in anti-fatigue treatments, showing good application prospects [21,22].

Currently, people are seeking new types of functional health drinks that can improve exercise capacity and relieve fatigue without side effects. According to reports, both ginseng and GBR contain nutrients with anti-fatigue effects, such as GABA [23] and ginsenoside [24]. Previous studies have demonstrated that fatigue is a complex and multidimensional symptom that varies in severity among individuals and affects daily life [25]. According to Identified Food Material Medica, ginseng rice congee has long been considered a prescription cure for consumptive diseases. Modern medicine posits that fatigue syndrome is a wasting disease. Ginseng and GBR, as ingredients in Korean snack recipes, have significant hypoglycemic, oxidative stress, and antioxidant effects and may show good effects in the development of anti-fatigue products [26]. Moreover, fermented puree has gradually become a popular food for consumers. Studies have shown that fermentation technology can not only reduce economic costs, but also improve food flavor. Based on retaining the original nutritional components of brown rice, it can also enrich the types of brown rice foods, improve their edible qualities, and make brown rice easier to digest by the human body, thereby improving bioavailability [27,28,29]. It is well known that probiotics are involved in the fermentation process, and these probiotics have a series of functional properties such as regulating the intestines [30]. Interestingly, different strains can produce different flavors after fermentation and increase the content of specific active ingredients, such as GABA and rare ginsenosides [31,32]. However, the changes in the active ingredients and the effects of the fermentation of beneficial microorganisms on the anti-fatigue activity of compound purees have not been examined.

Therefore, this study aimed to investigate the possibility of using ginseng and GBR to develop a novel herbal sports drink to attenuate fatigue. To our knowledge, this is the first study to explore the antioxidant and anti-fatigue effects of a fermented combination formula made from GBR and ginseng. In addition, this study is the first to report that the fermentation process can accelerate the conversion of rare ginsenosides and GABA simultaneously, thus advancing the development and utilization of GBR and ginseng.

## 2. Results

### 2.1. GABA Content

GABA, a non-protein amino acid, is one of the important inhibitory neurotransmitters in the mammalian nervous system and directly affects sensations of pain and anxiety [33]. Studies have reported that lactic acid bacteria are potential GABA-enriching microbiota, and GBR fermented using lactic acid bacteria could enrich the content of GABA, further enhancing its biological functions [34]. The effect of fermentation on the GABA content in the ginseng and GBR compound puree is shown in Table 1 (Figure S1). It is obvious that fermentation contributed markedly to the level of GABA in the compound drinks. The GABA content increased from 0.86 mg/100 mL before fermentation to 2.27 mg/100 g after, which is a 2.64-fold increase. To make up for the deficiency of pure inoculation fermentation, a mixture of four species of lactic acid bacteria were selected to optimize the fermentation conditions for GABA accumulation in the puree formulated in the study.

### 2.2. Ginsenoside Content

The biotransformation of ginsenosides using lactic acid bacteria has been well demonstrated in multiple studies. However, the biotransformation of ginsenosides in formulated purees through microbial fermentation has not been sufficiently investigated. In this study, we investigated the changes in the composition of ginsenosides in our formulated puree before and after fermentation (Table 2, Figure S2). Our data showed that the contents of ginsenosides Rg_1_, Re, Rf, Rb_1_, Rc, Rg_2_, Rd, and Rg_6_ decreased after co-fermentation, while the contents of ginsenosides Rh_4_, Rg_3_, and CK increased significantly. 

**Figure 1:**
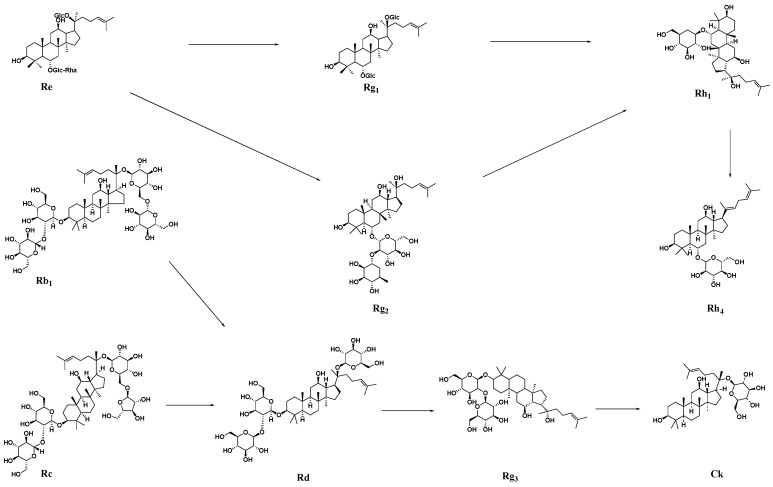
Transformation pathway of ginsenoside in FGB.

This may be due to the transformation of prototype ginsenoside in the ginseng extract by probiotic fermentation, which makes it produce rare ginsenosides with better activity. The ginsenoside Re can be transformed into Rg_1_ and Rg_2_ under the action of probiotic fermentation. With continuous fermentation, Rg_1_ and Rg_2_ continue to be transformed into Rh_1_ and its final dehydrated product, Rh4. The ginsenosides Rg_3_ and CK are derived from the fermentation transformation of Rc and Rb_1_, resulting in Rd. With continuous fermentation, the rare saponins Rg_3_ and CK are finally transformed (Figure 1).

### 2.3. In Vitro Antioxidant Activity of Different Purees

Both fermentation technology and formulation selection altered the antioxidant capacity of the purees (Table 3). In this study, the DPPH radical scavenging activities varied significantly among the different fermented formulation purees, ranging from 51.99% to 79.18%. FGB, FB, and FG showed antioxidant activities of 79.18%, 75.57%, and 74.39%, respectively, which were slightly lower than that of 200 μg/mL vitamin C as a positive control. In addition, the FRAP values of FGB and NFGB (33.60 and 27.93 mg FE/g, respectively) were higher than purees using ginseng or GBR alone (*p* < 0.05).

### 2.4. Cytotoxicity of Puree and H_2_O_2_

**Figure 2:**
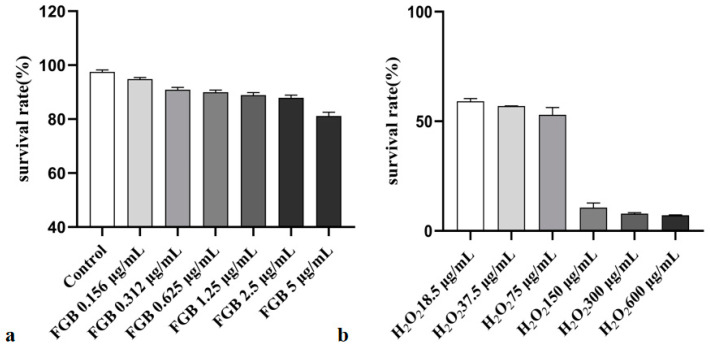
Inhibitory activity of FGB and H_2_O_2_ on proliferation of HepG_2_ cells (24 h). (**a**). determination of activity of FGB on HepG2 cells; (**b**). H_2_0_2_ activity determination of HepG2 cells.

In this experiment, 0.15625–5 μg/mL of fermented ginseng puree with GBR was selected for toxicity testing. The survival rate of the control group was 100% as a standard, and the appropriate safe concentration was screened for subsequent experiments. The results are shown in Figure 2. When the concentration of the puree was in the range of 0–2.5 μg/mL, it had no significant effect on the proliferation inhibition activity of HepG_2_ cells. In subsequent experiments, puree in the range of 1.25–2.5 was used. Subsequently, H_2_O_2_ (18.5–600 μg/mL) was used to determine the proliferation inhibition activity of HepG_2_ cells. It was found that when H_2_O_2_ (18.5 μg/mL) was used, the inhibition rate of the HepG_2_ cells reached 42%. When the concentration was further increased to 75 μg/mL, the cell proliferation inhibition activity did not change significantly. Subsequently, H_2_O_2_ (18.5 μg/mL) was used for modeling.

### 2.5. Effect of Puree on H_2_O_2_-Induced ROS Release in HepG_2_ Cells

**Figure 3:**
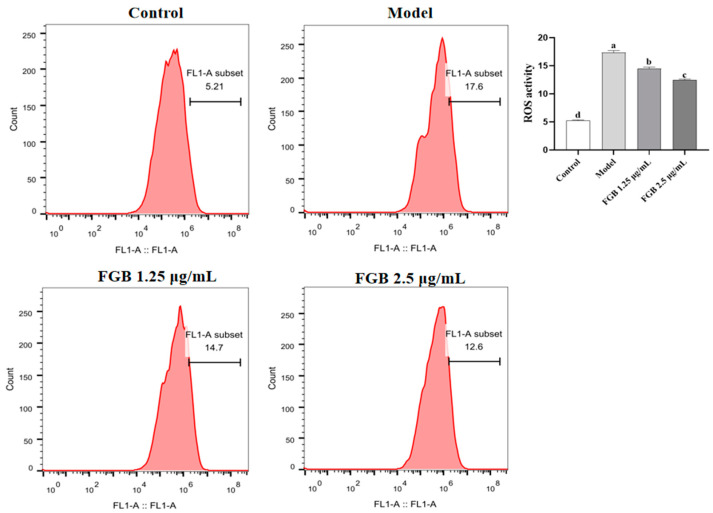
Effects of FGB and H_2_O_2_ on ROS release in HepG_2_ cells. Data are presented as mean ± SD (*n* = 3). Different letters on the bar are significantly different (*p* < 0.05).

The generation and clearance of ROS in normal cells are in a dynamic equilibrium state. Appropriate reactive oxygen species can promote macrophages to perform the immune physiological functions of phagocytosis and enzymatic hydrolysis. However, when stimulated by oxidation, as shown in Figure 3, the regulatory mechanism of intracellular ROS clearance is disordered, and excessive accumulation leads to oxidative damage and cell apoptosis. When the cells were subjected to H_2_O_2_-induced oxidative damage, the intracellular ROS level increased significantly compared with the control group. The ROS level significantly reduced after treatment with different concentrations of FGB. This shows that FGB can significantly reduce the increase in ROS levels in HepG_2_ cells caused by H_2_O_2_ (*p* < 0.05), inhibit the oxidative damage to cells caused by ROS, and play a role in antioxidative stress activation.

### 2.6. Effect of Puree on the Mitochondrial Membrane Potential of HepG_2_ Cells Induced by H_2_O_2_

**Figure 4:**
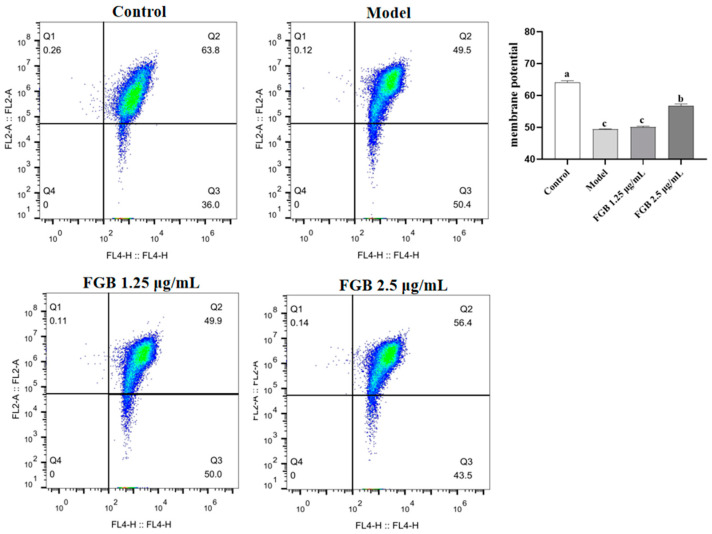
Effects of FGB and H_2_O_2_ on mitochondrial membrane potential of HepG_2_ cells. Data are presented as mean ± SD (*n* = 3). Different letters on the bar are significantly different (*p* < 0.05).

**Figure 5:**
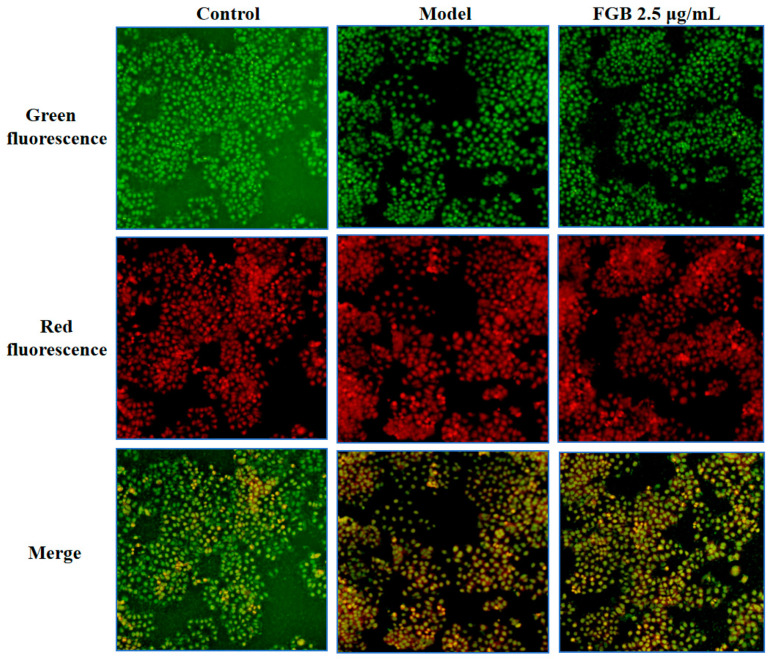
Effects of FGB on H_2_O_2_-induced acridine orange in HepG_2_ cells (×200).

The mitochondrial membrane potential results are shown in Figure 4. After H_2_O_2_ induction, the mitochondrial membrane potential of HepG_2_ cells decreased, indicating that after H_2_O_2_ treatment, 5,5′, 6,6′-Tetrachloro-1,1′, 3,3′–tetraethyl-imidacarbocyanine (JC-1) aggregates decreased, JC-1 monomers increased, red fluorescence decreased, and green fluorescence increased, indicating that H_2_O_2_ can induce changes in mitochondrial membrane potential through the mitochondrial pathway, thereby promoting cell apoptosis. After FGB intervention, the mitochondrial membrane potential increased, and increasing the FGB reduced the decrease in mitochondrial membrane potential induced by H_2_O_2_ by reducing the generation of ROS (Figure 4). Further, we investigated the effect of FGB on H_2_O_2_-induced HepG_2_ cell damage through acridine orange staining. The experimental results showed that in the H_2_O_2_-induced model group, the yellow-green fluorescence of HepG_2_ cells increased significantly, indicating that H_2_O_2_ induced oxidative damage to HepG_2_ cells, leading to cell apoptosis. After FGB drug intervention, the yellow-green fluorescence decreased significantly, and increasing FGB had a good antioxidant effect and could improve H_2_O_2_-induced cell damage (Figure 5).

### 2.7. Anti-Fatigue Activity

#### 2.7.1. Effects of Puree on Body Weight and Food Intake in Mice

**Figure 6:**
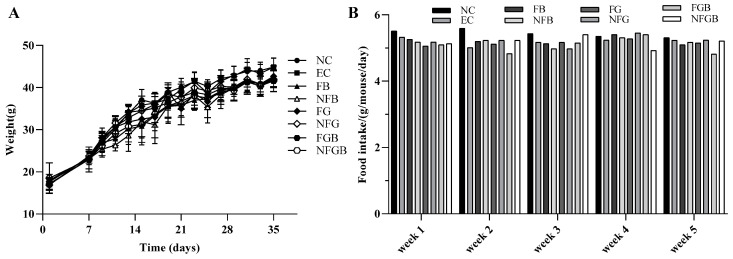
Effects of different purees on body weight (**A**) and food intake (**B**) of mice. Data are presented as mean ± SD (*n* = 10). *p* < 0.05 indicates significant difference.

The effect of the puree on body weight and food intake was recorded before the experiment (initial) until the 35th day (final). As presented in Figure 6A,B, in addition to normal increases, no significant difference was observed in the body weights and food intake of the mice between the control group and each treatment group (*p* > 0.05). This suggested that the safety of the puree was sufficient.

#### 2.7.2. Effects of Different Purees on Exhaustive Swimming Time

**Figure 7:**
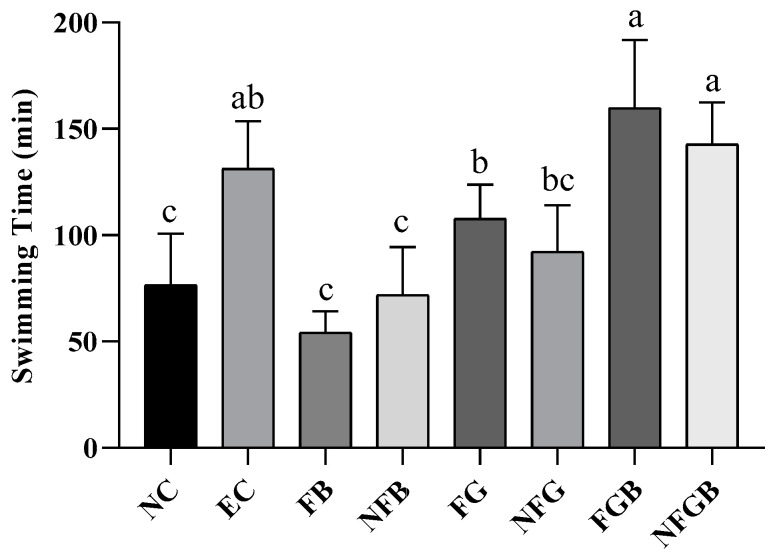
Effects of different purees on the weight-loaded forced swimming time in mice. Data are presented as mean ± SD (*n* = 10). Different letters on the bar are significantly different (*p* < 0.05).

The improvement in exercise endurance was the most direct and objective indicator of enhanced anti-fatigue ability. The length of the exhaustive swimming time indicated the degree of fatigue [35]. As shown in Figure 7, the exhaustive endurance levels in mice administered FGB and NFGB were 159.7 ± 32.0 and 142.8 ± 19.6 min, respectively. Compared to the NC group, the EC group of mice had a significantly prolonged forced swimming time (*p* < 0.05). The exhaustive swimming times of the FGB and NFGB groups were slightly longer than for the EC group (*p* > 0.05), increasing by 21.63% and 8.76%, respectively.

#### 2.7.3. Effects of Different Purees on Blood Urea Nitrogen (BUN) and Creatinine (CRE) 

**Figure 8:**
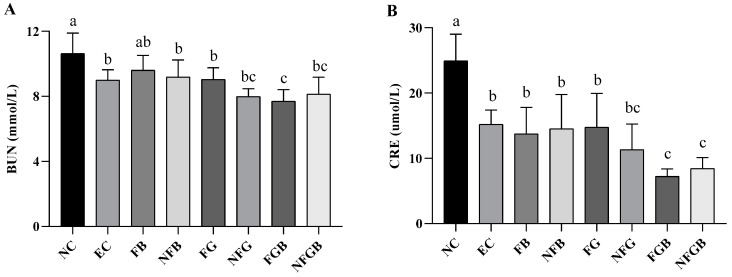
Effects of different purees on blood urea nitrogen (**A**) and creatinine (**B**) in mice. Data are presented as mean ± SD (*n* = 10). Different letters on the bar are significantly different (*p* < 0.05).

BUN was one of the essential indicators used to evaluate exercise endurance and fatigue status [36]. Figure 8A depicts the differences in BUN concentration after swimming. Compared with the NC group, the level of BUN decreased in all groups. The BUN content in the FGB groups was significantly lower than that of the EC group and decreased by 14.35% (*p* < 0.05). The BUN levels were slightly lower in the single unfermented puree groups compared to the single fermented puree groups (*p* > 0.05).

The excessive accumulation of CRE, which is the product of muscle metabolism in the body, can lead to fatigue [37]. The effect of different purees on CRE levels in mice is presented in Figure 8B. The CRE content in all groups was significantly lower than that in the NC group (*p* < 0.05). In addition, there was a significant difference in the CRE content between the FGB and NFGB groups compared to the EC group (*p* < 0.05). The results indicated that the combination of ginseng and GBR significantly accelerated the elimination of muscle metabolites. 

#### 2.7.4. Effects of Different Purees on Blood Lactic Acid (BLA) and Lactate Dehydrogenase (LDH) 

**Figure 9:**
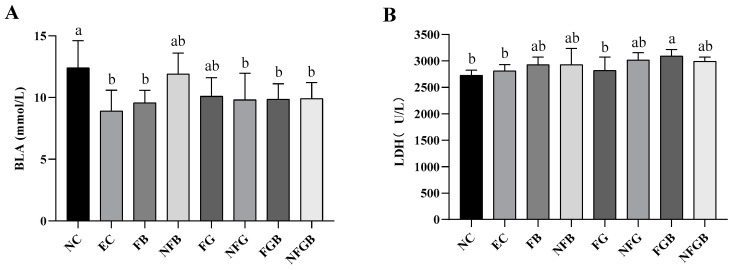
Effects of different purees on blood lactic acid (**A**) and lactic dehydrogenase (**B**) in mice. Data are presented as mean ± SD (*n* = 10). Different letters on the bar are significantly different (*p* < 0.05).

As shown in Figure 9A, the BLA levels were significantly reduced by swimming training in the EC group compared to the NC group. Moreover, the BLA levels of FB, NFG, FGB, and NFGB were both significantly lower than that of the NC group (*p* < 0.05) with decreases of 22.87%, 20.82%, 20.51%, and 20.08%, respectively. Our data suggested that the GBR fermented puree resulted in a highly significant decrease in the BLA of mice compared to the unfermented puree. In this study, the LDH activity levels among all groups of mice except the FG group were higher than the EC group (Figure 9B). The LDH activity of mice administered FGB increased by 9.93% compared with the EC group.

#### 2.7.5. Effects of Different Purees on Glycogen Storage

**Figure 10:**
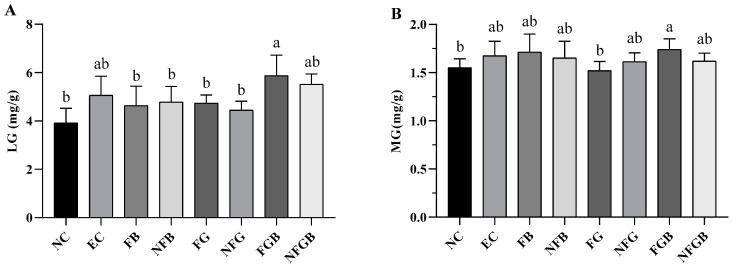
Effect of different purees on glycogen accumulation of liver (**A**) and muscle (**B**) in exhausted mice. Data are presented as mean ± SD (*n* = 10). Different letters on the bar are significantly different (*p* < 0.05).

Figure 10A,B depict the effects of different purees on the LG and MG in mice after swimming. The EC group showed high glycogen levels, but there was no significant difference between the NC and EC groups (*p* > 0.05). Compared to the NC group, the LG content in the FGB and NFGB groups increased by 49.71% and 40.51% (*p* < 0.05), respectively (Figure 10A). A significant increase in the MG content of 12.25% was observed in the FGB group relative to the NC group (*p* < 0.05). 

#### 2.7.6. Effect of Different Purees on Antioxidant Activity In Vivo

**Figure 11:**
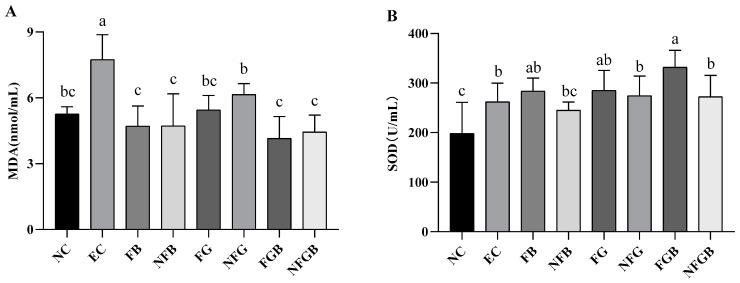
Effect of different purees on malondialdehyde (**A**) and superoxide dismutase (**B**) in mice. Data are presented as mean ± SD (*n* = 10). Different letters on the bar are significantly different (*p* < 0.05).

The levels of malondialdehyde (MDA) and superoxide dismutase (SOD) in the serum of exhausted swimming mice are shown in Figure 11A. Similar to the previous study, swimming exercise significantly increased the MDA level in serum in the EC group compared to that of the NC group [38]. Compared with the EC group, the MDA content in the FB, NFB, FGB, and NFGB groups decreased by 39.19%, 39.10%, 46.43%, and 42.62%, respectively (*p* < 0.05). Furthermore, supplementation with different purees significantly increased SOD activity after exhaustive swimming exercise (Figure 11B). The SOD activity of the FGB group was significantly higher than that of the EC group (*p* < 0.05) and increased by 26.50%. 

## 3. Discussion

Fatigue has become a principal problem that seriously affects physical and mental health and reduces work efficiency and quality of life. Indeed, a variety of anti-fatigue nutrition drinks used today to improve anti-fatigue have some adverse effects, which has driven us to develop a functional health puree using natural ingredients [39]. In this study, for the first time, six purees were formulated using ginseng and GBR that had previously been fermented or unfermented using probiotic bacteria to evaluate the effects of fermentation and combined application on antioxidant and anti-fatigue activities.

In recent years, fermentation has been considered an effective method to improve the antioxidant activity of purees [40]. Compared with other unfermented or single-fermented puree, the FGB puree prepared using co-fermentation technology showed better in vitro and in vivo antioxidant effects and in vivo anti-fatigue activities. This may be caused by the fermentation technology converting the original active substances in germinated brown rice or ginseng extract into active substances with better antioxidant and anti-fatigue activities. GABA, also known as aminobutyric acid, is a natural bioactive component in animals and plants [31]. GABA plays an important role in anti-fatigue, which helps the body recover after strenuous exercise and reduces the fatigue degree of the body. Grain products, dairy products, and beverage products rich in GABA have promising prospects in the research and development of functional foods to relieve physical fatigue [31]. Zhang et al. found that fermented milk prepared with the GABA high-yielding strain Lactobacillus brevis ATCC 367 can produce the highest GABA content and shows a strong anti-fatigue effect in mice. This mainly manifests in inhibiting blood lactate, urea nitrogen, and lactate dehydrogenase content; increasing liver glycogen, muscle glycogen, and ATP levels; and reducing reactive oxygen levels by increasing the concentration of SOD and peroxidase and inhibiting the content of malondialdehyde. Moreover, it stimulates the expression of adenosine 5′-monophosphate (AMP)-activated protein kinase (AMPK) and PPARγ coactivator-1α (PGC-1α) in muscles to relieve fatigue [41]. It is suggested to add 1–4% in drinks, coffee, tea, and other drinks, which can be used as functional products to relieve physical fatigue of people [42]. In our study, we found that the content of GABA in NFGB products was 0.86%, and after fermentation, the content of GABA increased to 2.27%, with an increase of 163%. Meanwhile, in the exhaustive swimming test, both FGB and NFGB showed significant anti-fatigue effects in mice, and the exhaustive swimming time was increased by 21.63% and 8.76% respectively. FGB showed a more potent anti-fatigue effect than NFGB. This result can also indirectly indicate that the higher level of GABA may be the main reason for the more potent anti-fatigue effect. 

Ginsenosides in ginseng extracts may also be the main active ingredient in FGB puree. Among them, the ginsenoside Rg_1_ has a good improvement effect on chronic fatigue, sports fatigue, and immune fatigue [18,19,43]. In an in-depth study of ginsenosides in terms of their anti-fatigue activity, it was found that rare ginsenosides, such as Rg_3_, CK, and Rh_4_, showed better anti-fatigue activity. Studies have shown that Rg_3_ can enhance anti-fatigue and antioxidant effects by activating the silent information regulator of transcription [44]. Lan et al. investigated the effects of the ginsenoside CK on the anti-fatigue effect of exhausted swimming rats and on skeletal muscle oxidative stress [45]. The results showed that, compared with the exercise model group, the rats in the low-, medium-, and high-dose CK groups exhibited increased exhaustive swimming time, muscle glycogen and liver glycogen reserve levels, and NF-E2-related factor 2 (Nrf2) or heme oxygenase-1 (HO-1) protein expression, and decreased BLA, BUN, and skeletal muscle MDA levels, suggesting that CK may activate the Nrf2/HO-1 signaling pathway, thereby enhancing the anti-oxidative stress capacity of skeletal muscle and producing anti-fatigue effects [45]. Interestingly, ginseng root extract residue as a fermentation medium for Ganoderma lucidum increased the content of the ginsenosides Rg_3_ and CK, which may be caused by the conversion of Rc during the fermentation process [46]. Rh4 may be derived from the conversion of Rg_1_, Rg_2_, Re, and Rf [47]. Our data showed that after co-fermentation, the contents of prototype ginsenosides Rg_1_, Re, Rf, Rb_1_, Rc, Rg_2_, Rd, and Rg_6_ in the puree decreased, while the contents of the rare ginsenosides Rh_4_, Rg_3_, and CK increased significantly, indicating that co-fermentation can promote the further conversion of prototype saponins in the puree into rare ginsenosides with better activity.

Following this, we used FRAP and DPPH free radical scavenging experiments to analyze the antioxidant capacity of the six purees. According to our results, the antioxidant activity of the ginseng puree was significantly higher than the GBR puree, but lower than the ginseng–GBR co-fermented puree. This may be due to the high antioxidant activity of the active ingredients in ginseng. Similarly, the addition of ginseng extract during the fermentation process also improved the antioxidant activity of the puree [48]. Therefore, fermentation is one of the approaches that can be used to enrich antioxidant activity. Then, we established an oxidative stress model of HepG_2_ cells induced by H_2_O_2_ and further evaluated the in vitro antioxidant activity of GBR puree. The results showed that GBR puree had a significant reversal effect on H_2_O_2_-induced ROS generation and membrane potential reduction in HepG_2_ cells, and acridine orange staining also showed that GBR significantly improves H_2_O_2_-induced cell damage. The antioxidant activity of GBR puree at the cell-free or cellular level indicates the advantages of developing functional puree to relieve fatigue. A good antioxidant status helps improve the immune function of the body and resist disease invasion, which is especially important for athletes who often perform high-intensity training. Proper antioxidant support can also help to prevent tissue damage caused by overtraining and accelerate the repair process, thus relieving fatigue. Taking exogenous anti-fatigue supplements has always been the main way to relieve fatigue.

Further, we evaluated the potential of GBR in terms of anti-fatigue effects. The weight-loaded forced swimming test was used to evaluate the anti-fatigue effects in mice. In this study, the swimming exercise training program was conducted on all mice for four weeks except the NC group, in which each puree was evaluated to improve endurance in the exercise training organism. We found that compound puree treatment synergistically prolonged the duration of exhaustive swimming in mice, while GBR or ginseng puree treatment alone resulted in no significant improvement compared with the EC group. These findings indicate that the combination of ginseng and GBR results in powerful anti-fatigue activity. Previous studies indicated that the combination of oyster peptide and ginseng extract treatment showed synergistic anti-fatigue effects by enhancing forced swimming endurance in mice [49].

For further exploration of the anti-fatigue ability of the different purees, several biochemical parameters derived from blood and tissues that contribute to fatigue were detected in the present study. High-intensity exercise can cause energy metabolism imbalance, increase protein and amino acid metabolism, and lead to the accumulation of BUN. Therefore, there is a positive correlation between urea nitrogen in vivo and exercise tolerance [50]. Endogenous creatinine is a product of muscle metabolism in the body and can be excreted through the kidneys. Hence, creatinine can reflect the ability of the kidneys to relieve fatigue [37]. It is noteworthy that both FGB and NFGB accelerated the elimination of BUN and CRE. However, the fermentation process had a more evident effect on BUN and CRE than that of the combination of ginseng and germinated brown rice, which might be related to the active components in the puree that accelerate the elimination of metabolites. With intense exercise, an increase in aerobic muscular activity could switch to anaerobic metabolism, leading to the accumulation of BLA and further altering the microenvironment, indirectly causing fatigue and other side effects [51]. Serum LDH maintains low activity in the blood in normal circumstances. When exercise increases, the skeletal muscle cell membranes may be damaged, leading to the penetration of LDH into the blood. Therefore, elevated LDH activity in the blood can accelerate the rate of lactate clearance from muscle, delaying the onset of fatigue or accelerating its elimination [52]. As expected, FGB displayed its anti-fatigue effect through the upregulation of LDH; however, the fermentation treatment had no significant effect on LDH activity. It was reported that protopanaxadiol-type and protopanaxatriol-type ginsenosides have been used to alleviate fatigue by inhibiting the accumulation of metabolites such as BLA and CRE induced by weight-loaded swimming, verifying that the anti-fatigue effects of ginseng were mainly attributed to its saponins, especially protopanaxatriol [53]. High levels of glycogen reserves provide more energy to maintain the homeostasis of blood glucose and prolong endurance exercise [54]. The data suggested that the ginseng and GBR puree had similar effects on LG and MG in exercise-fatigued mice. Interestingly, fermentation resulted in a significant increase in glycogen content in the combined ginseng and GBR puree compared to the puree containing a single plant, revealing a strong synergistic effect after compounding. High energy consumption during intense exercise may cause an imbalance between the oxidation and anti-oxidation systems, resulting in a reduction in antioxidant activities [55]. Antioxidant enzymes are regarded as the first line of defense against ROS, coordinated to reduce the generation of active oxygen radicals and prevent lipid peroxidation and intermediate products of metabolization from undermining bodies [56]. Our results indicated that the anti-fatigue effect of the different purees probably occurs through the protection of the corpuscular membrane by preventing lipid oxidation via modifying the activities of several enzymes. 

## 4. Materials and Methods

### 4.1. Materials and Reagents

The GBR was provided by Zhaixiang Ecological Agriculture Co., Ltd. (Benxi, China). Ginseng root extract with a yield of 28.53% was obtained from Runvo Biotechnology Co., Ltd. (Ningbo, China). *Streptococcus acidophilus*, *Lactobacillus acidophilus*, *Bifidobacterium bifidum*, and *Lactobacillus bulgaricus* were purchased from Puls International Trading Co., Ltd. (Tianjin, China). The ginsenosides Rg_1_, Rc, Rg_3_, CK, and Rh_2_ were previously isolated from the *Panax* genus by our laboratory (purity > 98%). The GABA was bought from Yuanye Biotechnology Co., Ltd. (Shanghai, China). The o-phthalaldehyde (OPA) and β-mercaptoethanol were obtained from Maclean Biochemical Technology Co., Ltd. (Shanghai, China). All of the chemicals and reagents were obtained from Tianjin Yongda Chemical Reagent Co., Ltd. (Tianjin, China).

### 4.2. Preparation of Puree

**Figure 12:**
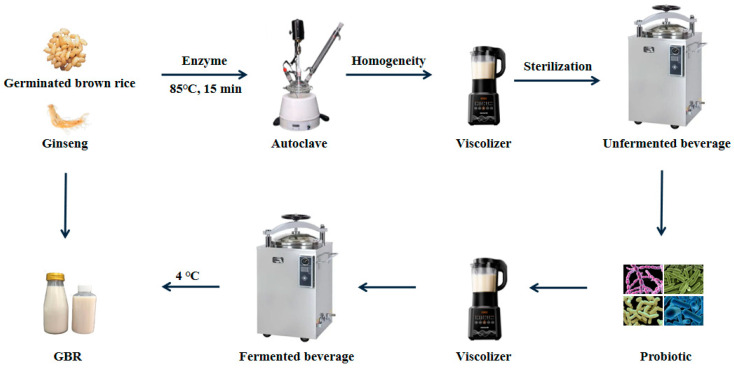
Schematic representation of puree processing and fermentation.

The puree was manufactured with GBR and ginseng extract. Briefly, a certain amount of GBR, ginseng extract, or the mixture was supplemented with three times the amount of water, heated until it gelatinized and achieved enzymatic hydrolysis, and then filtered. The resultant filtrates were homogenized, sterilized, and cooled to 37 °C. Then, *Streptococcus acidophilus*, *Lactobacillus acidophilus*, *Bifidobacterium bifidum*, and *Lactobacillus bulgaricus* were added for anaerobic static fermentation at 42 °C for 5 h. The filtrate was standardized through secondary blending, filtered, homogenized, and sterilized. Finally, the puree was placed into a sterile bottle and stored under refrigeration at 4 °C until analysis (Figure 12).

### 4.3. Determination of GABA Content

The GABA content in the samples was determined using high-performance liquid chromatography (HPLC analysis was conducted on the L-2200 HITACHI HPLC system equipped with a HITACHI 1430 DAD detector, 1310 column incubator, 1210 injection tray, and 1110 pump) (Hitachi High-tech Company, Tokyo, Japan). The puree samples were processed with acetonitrile to denature the protein, and the mixture remained at room temperature for 10 min and was centrifuged at 12,000 rpm for 10 min. Then, 200 μL supernatant was mixed with 1 mL OPA derivatizing reagent for 60 s. The derivatized samples were filtered for further analysis. An HPLC (Hitachi L 2000 Series, Hitachi, Tokyo, Japan) equipped with a Promosil C_18_ column (250 mm, 4.6 mm, 5 μm) was used for the content analysis. Both acetonitrile and 0.025 M sodium acetate (pH 5.90 ± 0.05) were used as the mobile phase with a constant flow rate of 1.0 mL/min. The injection volume was 10 μL. The absorbance was measured at 332 nm and the column temperature was 35 °C. The samples were independently analyzed in triplicate and the results were expressed as mg/100 mL.

### 4.4. Determination of Ginsenoside Content

Here, 1 g of sample was weighed accurately, and 75% ethanol solution was added (20 times). Ultrasonic-assisted extraction was performed for 30 min, repeated once, centrifuged at 4000 r/min for 10 min, and then the supernatant was collected. After centrifugation, the supernatant was concentrated to dryness under reduced pressure, and the residue was dissolved in 2 mL of chromatographic-grade methanol and filtered through a 0.45 μm organic microporous membrane into a sample injection vial with a built-in tube for analysis and detection.

The HPLC analysis was conducted on an L-2200 HITACHI HPLC system equipped with a HITACHI 1430 DAD detector, 1310 column incubator, 1210 injection tray, and 1110 pump (Hitachi High-tech Company, Tokyo, Japan). An Agela Promosil C_18_ column was also used (250 mm × 4.6 mm, 5 μm). The mobile phase A was acetonitrile solution, and the mobile phase B was aqueous solution. The volume flow rate was set to 1 mL/min, the column temperature was 35 °C, the absorbance was 203 nm, the injection volume was 20 μL, and the elution gradient process was 0 min, 20% A; 0–20 min, 20% A; 20–45 min, 20–46% A; 45–55 min, 46–55% A; 55–60 min, 55%A; 61–75 min, 55–20% A. Each sample was measured three times in parallel, the chromatogram was recorded, and the average value was calculated as the final result.

### 4.5. Antioxidant Activity Assay

#### 4.5.1. Measurement of Free Radical-Scavenging Activity (DPPH Assay)

DPPH radical scavenging activity was measured according to the previous method [57]. Briefly, 100 μL sample solution and 100 μL 0.1 mmol/L DPPH were mixed vigorously for 5 s and left to stand for 30 min at room temperature in the dark. Ascorbic acid was used as the positive control. All assays were carried out in triplicate. The absorbance was recorded at 540 nm. The scavenging activity was calculated using the following equation:

Scavenging activity (%) = (A_0_ − A/A_0_) × 100% (1)

DPPH solution plus sample solution.

#### 4.5.2. Measurement of Ferric-Reducing Antioxidant Power (FRAP Assay)

The FRAP assay was determined using the previous method with slight changes [58]. The FRAP reagent was prepared by mixing 10 volumes of 0.1 mol/L acetate buffer (pH 3.6) with 1 volume of 20 mmol/L FeCl_3_-6H_2_O and 1 volume of 10 mmol/L TPTZ solution and then kept in a water bath at 37 °C until use. Subsequently, 100 μL of the sample solution was added to 900 μL of FRAP reagent for 10 min at room temperature. The absorbance was measured at 593 nm. Ascorbic acid was used as the standard to establish a standard curve. The results were expressed as milligrams of the ascorbic acid equivalents’ antioxidant capacity. All assays were carried out in triplicate.

### 4.6. Effects of Fermented Puree of Ginseng with GBR and H_2_O_2_ on HepG_2_ Cells

#### 4.6.1. Cell Culture

HepG_2_ cells (SCSP-510) were purchased from the Chinese Academy of Science cell bank. The HepG_2_ cells were cultured in a 37 °C, 5% CO_2_ incubator in DMEM supplemented with 10% FBS (superior fetal bovine serum) and 1% penicillin–streptomycin.

#### 4.6.2. MTT Assay

To evaluate the effects of H_2_O_2_ and fermentation puree samples on the survival rate of HepG_2_ cells, an MTT assay was conducted. In summary, HepG_2_ cells were seeded into a 96-well plate (1.0 × 10^5^ cells per well) and allowed to grow to a cell density of around 80%. The HepG_2_ cells were treated with different concentrations of H_2_O_2_ (0, 37.5, 75, 150, 300, 600 μM) for 20 h. After treatment, 10 μL of MTT solution was added to each well and cultured for another 4 h. Then, the culture medium was discarded from the wells and 100 μL of DMSO solution was added inward. It was allowed to stand for 20 min and then the absorbance values of each well were measured, the drug inhibition rate was calculated, and the H_2_O_2_ induction dose was determined based on the cell inhibition rate. As above, HepG_2_ cells were treated with different concentrations of puree samples (0, 0.625, 1.25, 2.5, 5, and 10 μg/mL), and the drug inhibition rate was calculated to determine the safe dosage of the puree samples.

#### 4.6.3. Detection of Oxidative Stress Level and Mitochondrial Membrane Potential

HepG_2_ cells were inoculated in a 6-well plate and were induced using the puree samples (1.25, 2.5 μg/mL) and H_2_O_2_ (37.5 μg/mL) for 24 h. After induction, the oxidative stress level and mitochondrial membrane potential of the HepG_2_ cells were detected according to the instructions of the kit.

ROS: The cell culture medium was removed, washed with PBS solution, loaded with DCFH-DA (10 μM) in an in situ probe in serum-free DMEM medium, and washed twice with serum-free DMEM medium after loading. Then, the cells were collected in a flow cell tube, resuspended with PBS solution, and detected on the computer.

Mitochondrial membrane potential: The above-cultured cells were collected in 2 mL EP tubes and washed with PBS solution, and then JC-1 working solution was diluted 10 times with Incubation Buffer solution before being added to the EP tubes for incubation (500 μL per well). After incubation, the solution was washed twice with Incubation Buffer, and then each tube was resuspended with 500 μL Incubation Buffer and detected on the computer.

#### 4.6.4. Acridine Orange Strain

HepG_2_ cells were inoculated into a 6-well plate at an appropriate density and cultured for 24 h to allow them to adhere to the wall. Puree samples (1.25, 2.5 μg/mL) and H_2_O_2_ (37.5 μg/mL) were added to induce the HepG_2_ cells for 24 h. Then, the old culture medium was sucked off, washed with PBS solution three times, fixed with 4% paraformaldehyde for 15 min at room temperature, and washed three times with PBS solution. The cells were stained with acridine orange dye solution (10 mg/mL) for 30 s and washed with PBS solution three times. Then, the cells were washed with 0.1 Mol calcium chloride, left for 30 s, and washed several times with PBS. Finally, the well plate was placed under an inverted fluorescence microscope for observation, and the image was observed, photographed with a 20-fold objective lens, and quantitatively analyzed using ImageJ 1.43.67 software.

### 4.7. Anti-Fatigue Activity Assay

#### 4.7.1. Mice and Exercise Protocol

Healthy Kunming mice (all males, 20 ± 2 g) were purchased from the experimental animal center of Shenyang Pharmaceutical University. The mice were housed in a suitable environment at room temperature (23 ± 2 °C) and under a moderate humidity level of 55 ± 5% with a 12 h day/night circadian rhythm. Before the experiments, the mice were adaptively fed for at least one week. All of the experimental procedures were conducted in accordance with the Guide for the Care and Use of Laboratory Animals (Approval protocol number: SYPU-IACUC-2021-1-6-105). 

After a week of acclimatization, the mice were randomly divided into eight groups: normal control group (NC), exercise control group (EC), fermented germinated brown rice group (FB), unfermented germinated brown rice group (NFB), fermented ginseng group (FG), unfermented ginseng group (NFG), fermented germinated brown rice and ginseng group (FGB), and unfermented germinated brown rice and ginseng group (NFGB). In each group, 10 mice were used for weight-loaded forced swimming to determine the swimming time, and the remaining 10 mice were used for collecting blood, liver, and gastrocnemius muscle to determine the fatigue-related biochemical parameters after forced swimming for 90 min. The exercise groups were given the purees via oral administration every day between 9:00 and 10:00 a.m. and the NC was given the same amount of distilled water. During 4 weeks of intragastric administration, the weight of mice was recorded every two days and food intake was noted daily. The mice in all groups except the NC group were trained for 30 min every three days. All animals had free access to diet and water throughout the entire experimental period.

#### 4.7.2. Exhaustive Swimming Test

Briefly, 30 min after the last treatment, the mice were forced to swim with a lead wire (5% of the mouse’s body weight) attached to their tail. Each mouse was dropped into a plastic swimming pool (50 cm × 50 cm × 40 cm) filled with water (28 ± 1 °C) to a depth of 30 cm. The exhausting time was immediately recorded when the mice sank into the water and failed to rise to the surface to breathe within 10 s.

#### 4.7.3. Determination of Biochemical Parameters

In the anti-fatigue activity evaluation, the rest of the mice without load were forced to swim in the abovementioned swimming pool for 90 min at the end of the 28th day. After resting for 30 min, the mice were anesthetized using 10% chloral hydrate in a ratio of 0.05 mL/10 g body weight through intraperitoneal injection, and blood samples were obtained via the classic enucleating eyeball collection method [59]. Then, the livers and gastrocnemius muscles of the mice were collected, immediately frozen in liquid nitrogen, and stored at −80 °C for further analysis. After centrifugation at 4000 rpm for 10 min at 4 °C, the serum samples were collected to determine the levels of BUN, BLA, CRE, LDH, SOD, and MDA. At the same time, the liver and gastrocnemius muscles were also taken to determine the content of liver glycogen (LG) and muscle glycogen (MG), respectively. All assay kits were purchased from Nanjing Jiancheng Bioengineering Institute Co., Ltd. (Nanjing, China).

### 4.8. Statistical Methods

All data were expressed as means ± SD. Statistical analyses were performed using GraphPad Prism 8.0.2 (GraphPad Software, San Diego, CA, USA). One-way ANOVA with Tukey’s test was used for inter-group comparison. *p*-values less than 0.05 were considered to be statistically significant. In this manuscript, the method of significant differences marked with letters is used. The highest column is given a value (a). When there is a group that is not significant with this group, the column corresponding to the corresponding group is marked with the same letter (a), and the significant column is not marked. The other columns are marked in alphabetical order. Different letters on the bar are significantly different (*p* < 0.05).

## 5. Conclusions

The co-fermented FGB puree was found to have better antioxidant and anti-fatigue activities than the unfermented puree. This may be related to the content of GABA and rare ginsenosides (Rh_4_, Rg_3_, and CK). Overall, we confirmed that ginseng and GBR can be used as new types of active antioxidant and anti-fatigue ingredients, and co-fermentation can effectively increase the content of active ingredients and relieve fatigue, which provides an important basis for the development and application of ginseng and GBR.

## Figures and Tables

**Table 1:** Chemical composition of the puree.

Compound	FGB	NFGB
GABA (mg/100 mL)	2.27 ± 0.12	0.86 ± 0.05
Data are presented as mean ± SD (*n* = 3).		

**Table 2:** Ginsenoside content of the puree.

Ginsenoside Content (mg/g)	Name			
	FG	NFG	FGB	NFGB
Rg1	0.17 ± 0.05	0.19 ± 0.02	0.41 ± 0.02	1.81 ± 0.13
Re	0.73 ± 0.02	0.56 ± 0.03	2.63 ± 0.12	3.73 ± 0.25
Rf	0.10 ± 0.01	0.28 ± 0.09	0.59 ± 0.13	0.77 ± 0.21
Rb1	1.39 ± 0.12	1.97 ± 0.13	7.22 ± 0.16	9.20 ± 0.36
Rc	8.00 ± 0.15	11.26 ± 0.34	6.12 ± 0.07	20.82 ± 0.32
Rg2	1.06 ± 0.07	1.33 ± 0.11	3.41 ± 0.18	5.74 ± 0.43
Rd	0.54 ± 0.01	0.62 ± 0.11	3.56 ± 0.05	4.73 ± 0.05
Rg6	0.01 ± 0.01	0.06 ± 0.01	0.37 ± 0.08	0.39 ± 0.04
Rh4	4.54 ± 0.11	2.75 ± 0.15	54.09 ± 1.51	21.66 ± 1.02
Rg3	0.07 ± 0.01	0.00 ± 0.01	0.11 ± 0.05	0.00 ± 0.00
CK	0.29 ± 0.01	0.00 ± 0.01	4.20 ± 0.11	0.00 ± 0.00

**Table 3:** In vitro antioxidant activity of different purees.

Sample	FRAP ^a^ (mg FE/g)	DPPH ^b^ (%)
fermented germinated brown rice group (FB)	18.51 ± 1.52	75.57 ± 1.29
unfermented germinated brown rice group (NFB)	15.10 ± 1.56	51.99 ± 1.43
fermented ginseng group (FG)	23.68 ± 1.25	74.39 ± 1.50
unfermented ginseng group (NFG)	19.90 ± 1.15	68.33 ± 1.53
fermented germinated brown rice and ginseng group (FGB)	33.60 ± 1.05	79.18 ± 3.51
unfermented germinated brown rice and ginseng group (NFGB)	27.93 ± 1.10	58.64 ± 2.08

^a^ Ferric-reducing antioxidant power. ^b^ Scavenging activity for the DPPH radical. Data are presented as mean ± SD (*n* = 3). FRAP is expressed as Fe^2+^ equivalents per g of the sample (mg FE/g sample).

## Data Availability

The original contributions presented in the study are included in the article. Further inquiries can be directed to the corresponding author.

## Supplementary Materials

The supporting information can be downloaded at: https://www.mdpi.com/article/10.3390/ijms251910359/s1.

## Abbreviation

γ-aminobutyric acid (GABA); fermented puree made of ginseng and germinated brown rice (FGB); Ferric ion-reducing antioxidant power (FRAP); 1,1-diphenyl-2-picryl-hydrazyl radical (DPPH); Brown rice (BR); Germinated brown rice puree (GBR); indoleamine 2,3-dioxygenase (IDO); 5,5′, 6,6′-Tetrachloro-1,1′, 3,3′–tetraethyl-imidacarbocyanine (JC-1); creatinine (CRE); blood lactic acid (BLA); lactate dehydrogenase (LDH); Malondialdehyde (MDA); superoxide dismutase (SOD); adenosine 5‘-monophosphate-activated protein kinase (AMPK) ; PPARγ coactivator-1 α (PGC-1α); NF-E2-related factor 2 (Nrf2); heme oxygenase-1 (HO-1); normal control group (NC); exercise control group (EC); fermented germinated brown rice group (FB); unfermented germinated brown rice group (NFB); fermented ginseng group (FG); unfermented ginseng group (NFG); unfermented germinated brown rice and ginseng group (NFGB); liver glycogen (LG); muscle glycogen (MG).
