# Supplementary material for: The Antioxidant and Anti-Fatigue Effects of Rare Ginsenosides and γ-Aminobutyric Acid in Fermented Ginseng and Germinated Brown Rice Puree
Source: Int J Mol Sci. 2024 Sep 26;25(19):10359. doi: 10.3390/ijms251910359 (PMC11476846; doi:10.3390/ijms251910359)
Supplement: Supplementary file 1 [file ijms-25-10359-s001.zip › ijms-3204043-supplementary.pdf]

Supporting information

The rare ginsenosides and γ-aminobutyric acid and anti-oxidant, anti-fatigue effects of the fermented puree of ginseng and germinated brown rice

Shiwen Feng[a †], Tao Li[a †], Xinrui Wei[b], Yifei Zheng[a], Yumeng Zhang[b], Gao Li [a *], Yuqing Zhao[a, b *]

[a] Key Laboratory of Natural Medicines of the Changbai Mountain, Ministry of Education, College of Pharmacy, Yanbian University, Yanji 133002, China

[b] Shenyang Pharmaceutical University, Shenyang 110016, China

* Corresponding author:

Yuqing Zhao: Tel: +8624 43520309; Fax: +8624 43520300; E-mail: Zyq2023@ybu.edu.cn

Gao Li：Tel: + 0433-2436001; E-mail: gli@ybu.edu.cn

[†] These authors contributed equally to this work

The supporting information includes:

Figure. S1. Chromatograms of RP-HPLC analysis of GABA in the fermented and non-fermented beverages from ginseng and germinated brown rice.

Figure. S2. Chromatograms of RP-HPLC analysis of ginsenosides in the fermented and non-fermented beverages from ginseng and germinated brown rice.

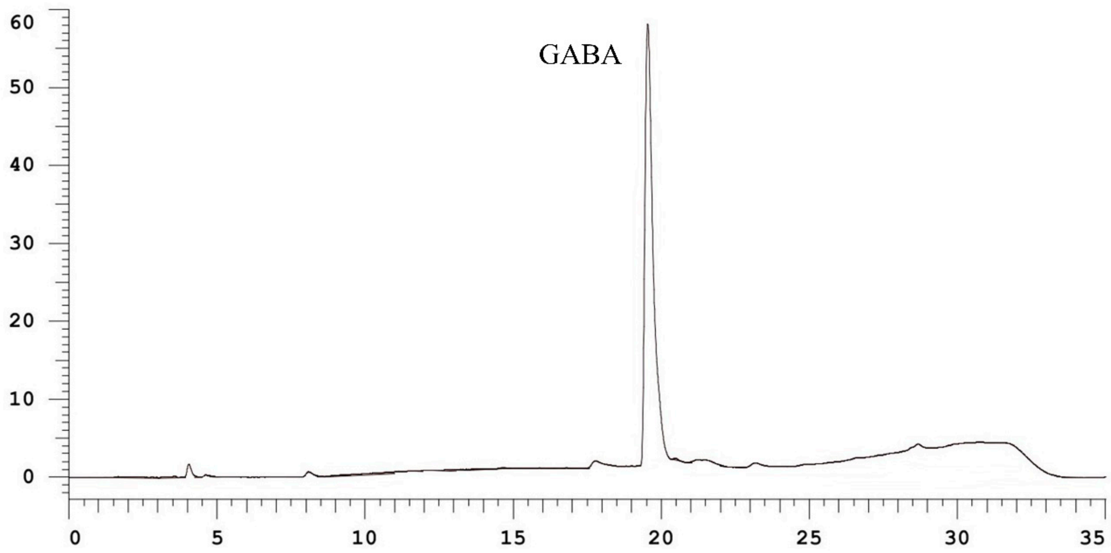

Figure S1A. GABA

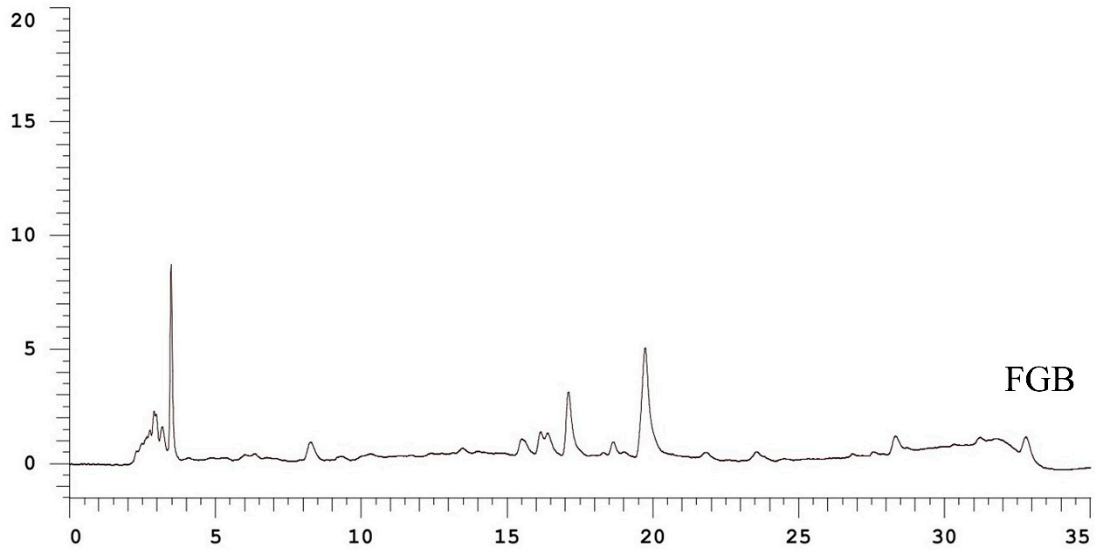

Figure S1B. FGB

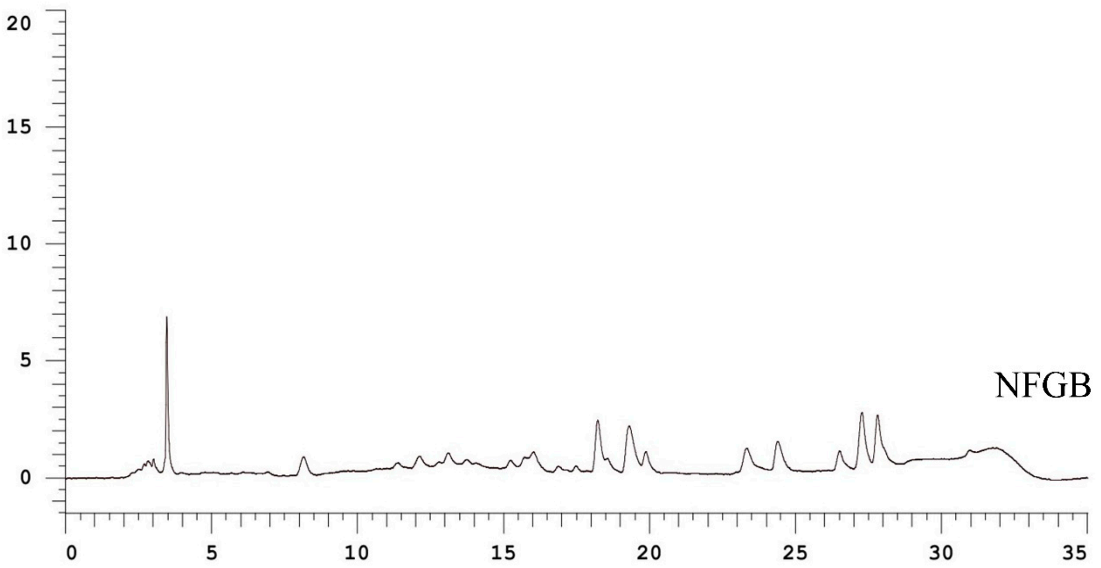

Figure S1C. NFGB

Figure S1. Chromatograms of RP-HPLC analysis of GABA in the fermented and non-fermented beverages from ginseng and germinated brown rice. A. GABA; B. FGB; C. NFGB.

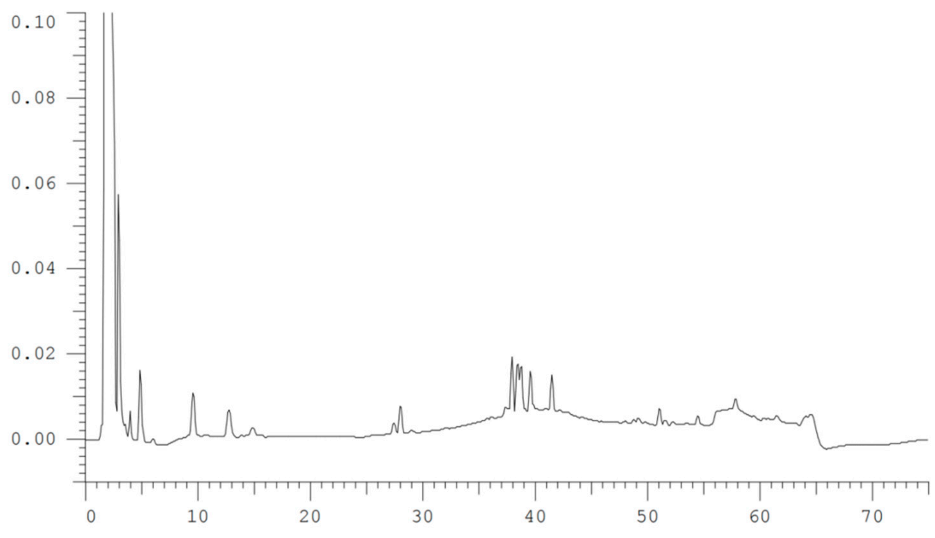

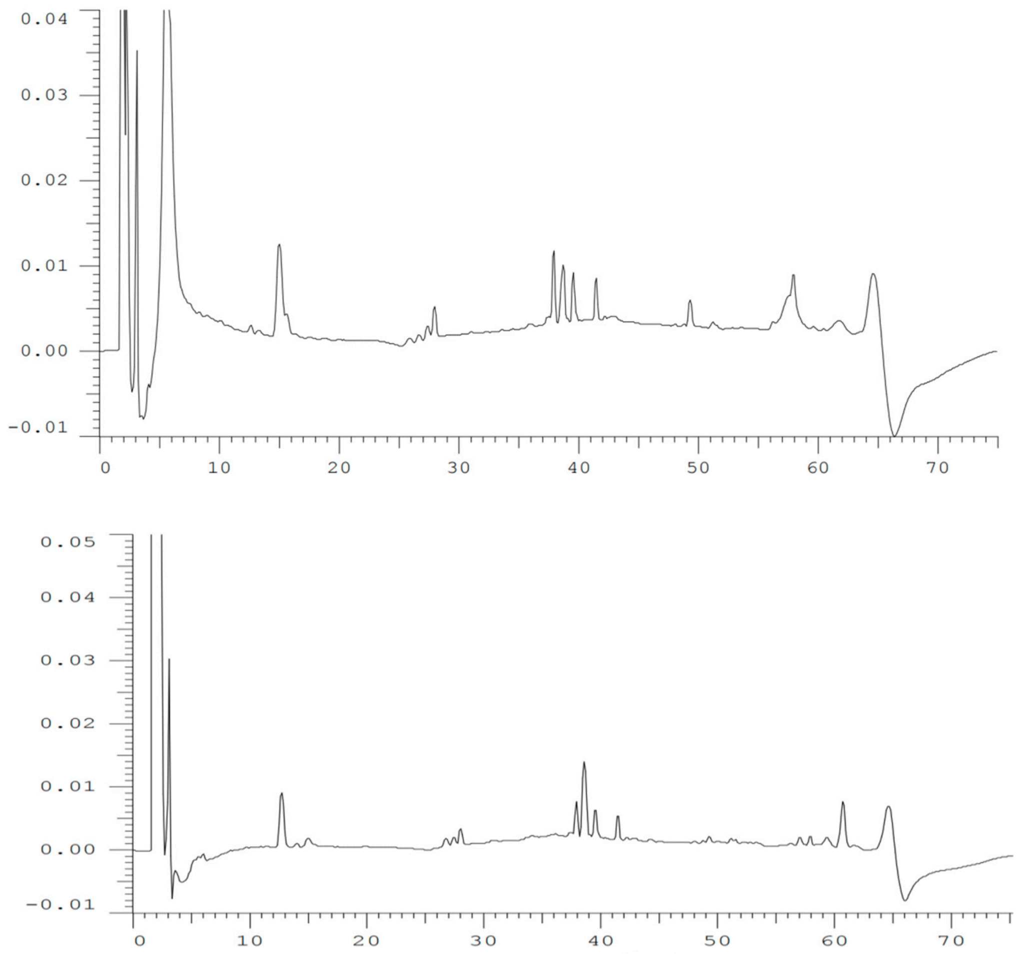

Figure S2A. NFG

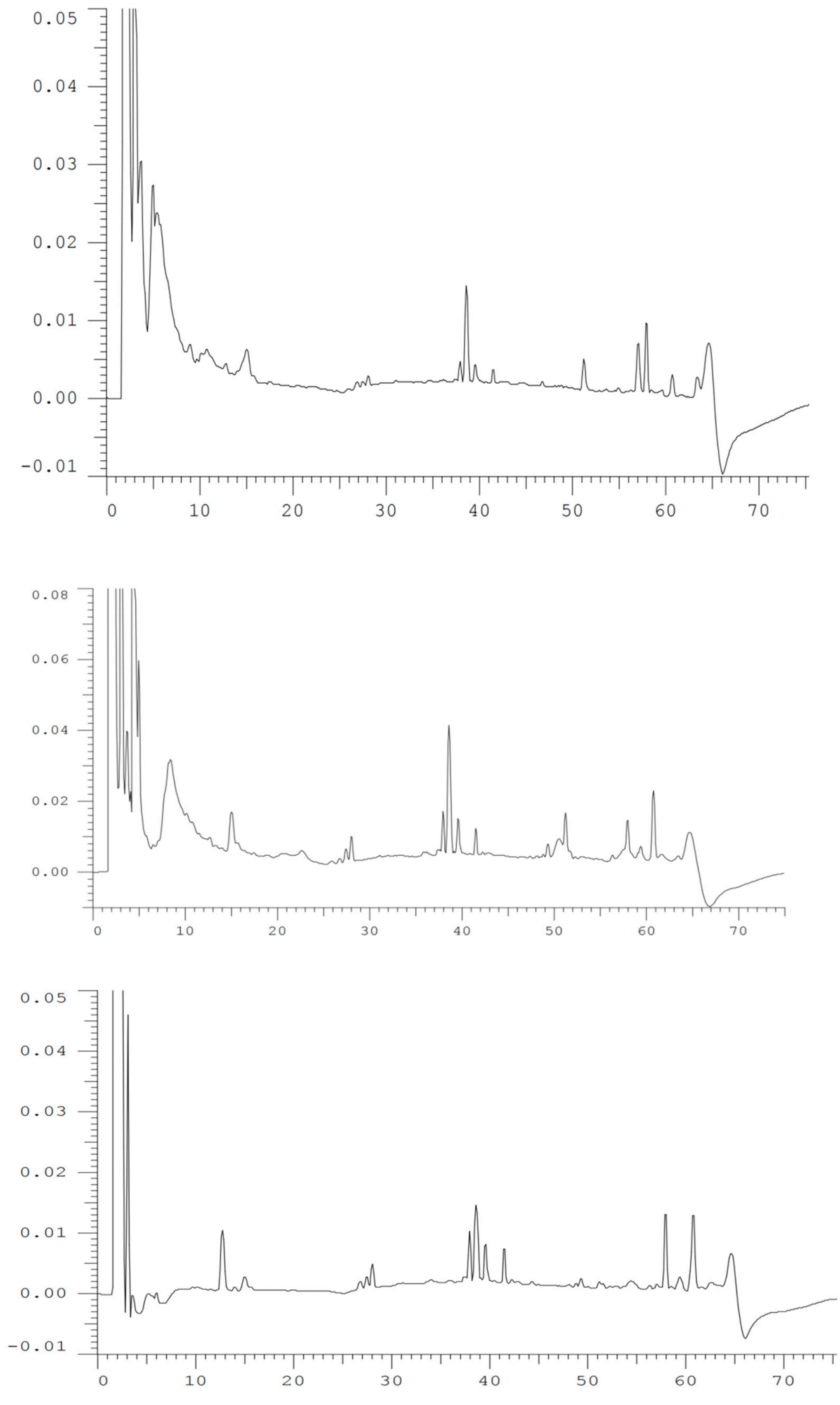

Figure S2B. FG

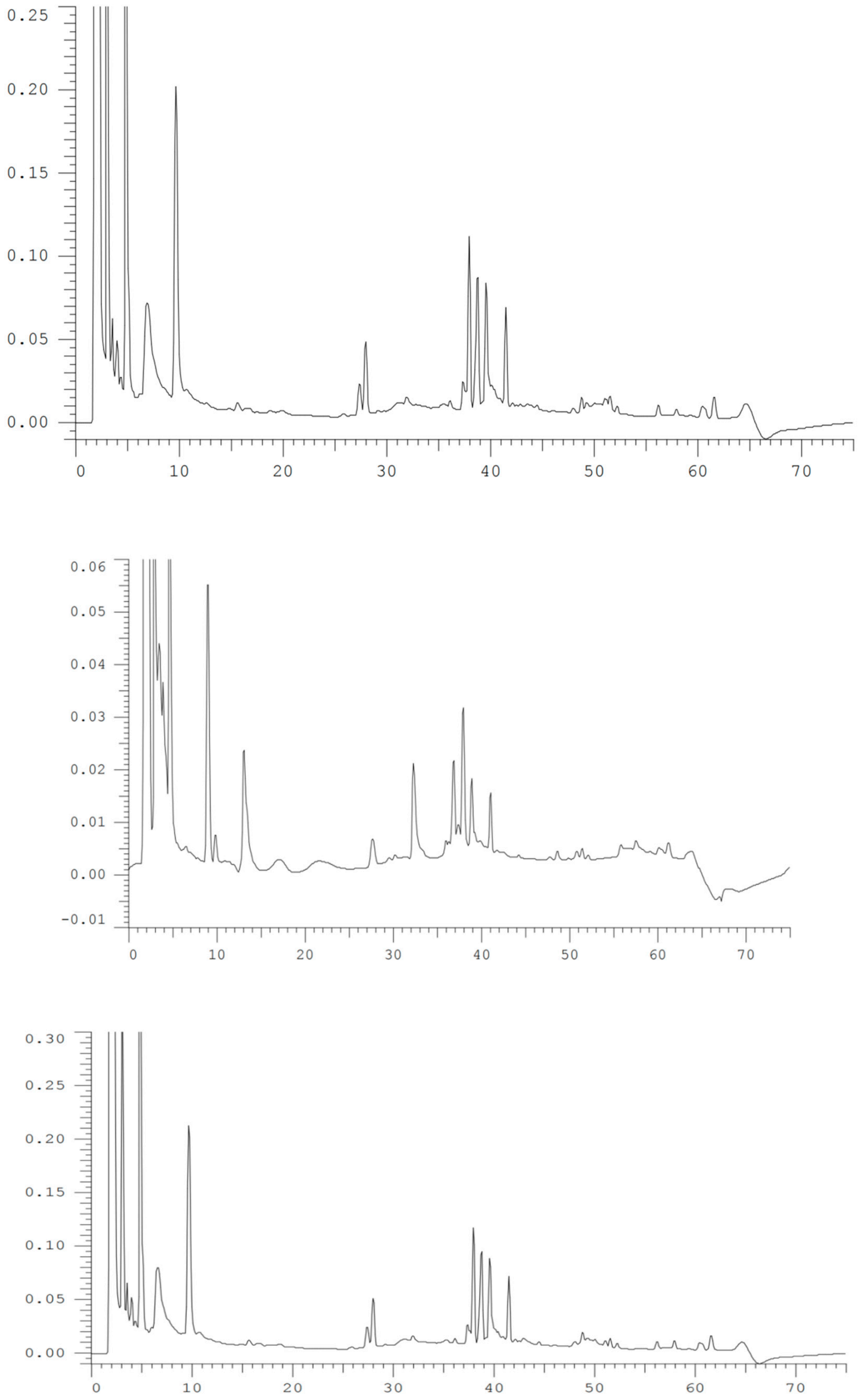

Figure S2C. NFGB

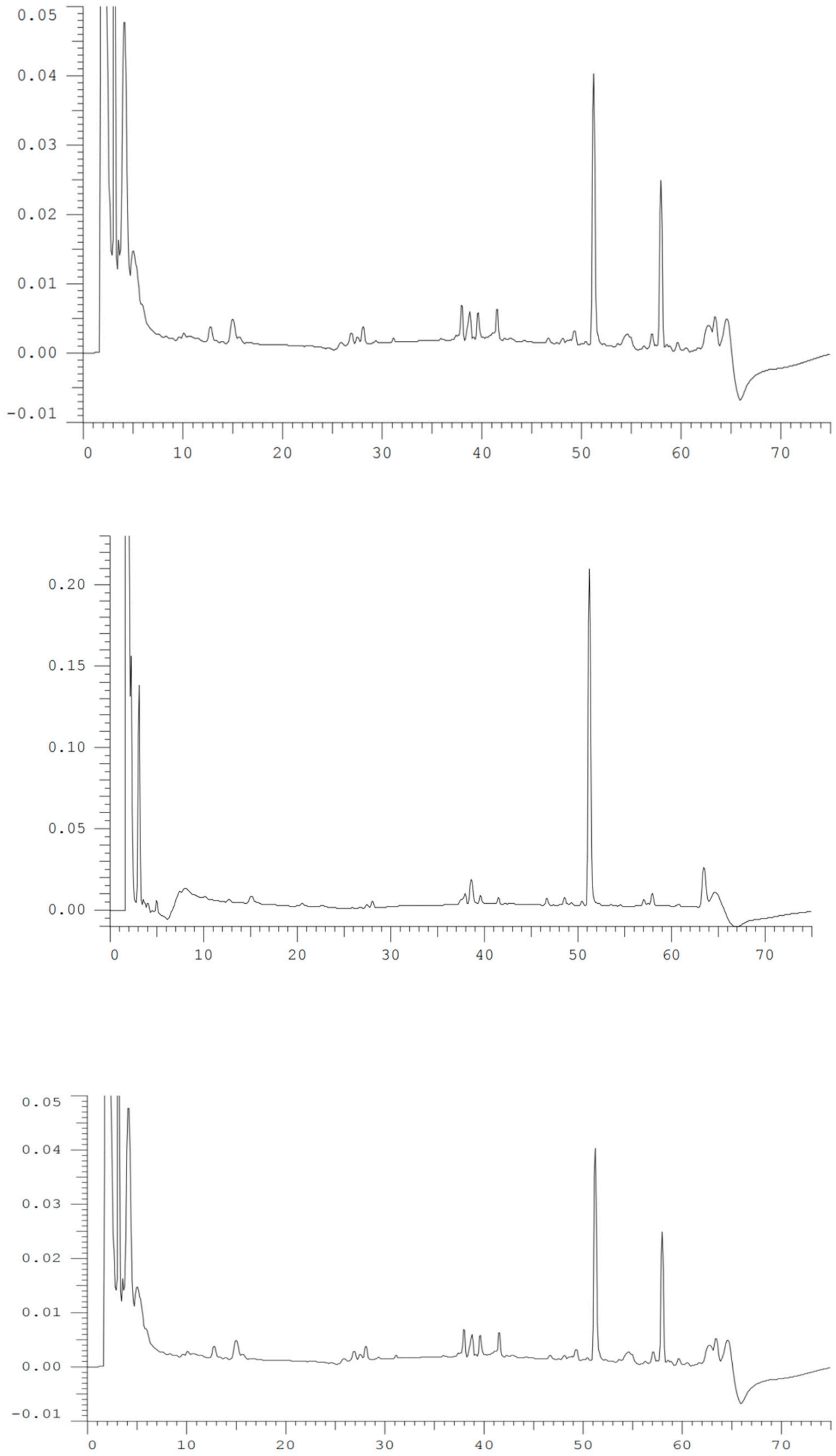

Figure S2D. FGB

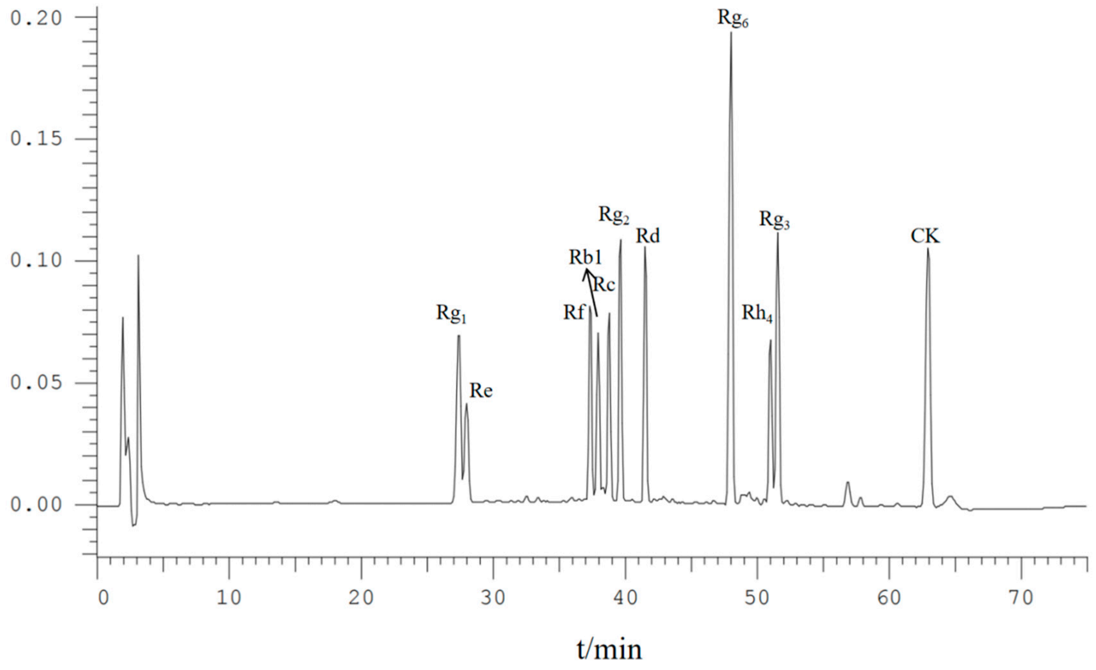

Figure S2E. Saponin standard

Figure S2. Chromatograms of RP-HPLC analysis of ginsenosides in the fermented and non-fermented beverages from ginseng and germinated brown rice. A. NFG; B. FG; C. NFGB; D. FGB; E. Saponin standard.
